# Review for the generalist: The antinuclear antibody test in children - When to use it and what to do with a positive titer

**DOI:** 10.1186/1546-0096-8-27

**Published:** 2010-10-20

**Authors:** Peter N Malleson, Murray J Mackinnon, Michaela Sailer-Hoeck, Charles H Spencer

**Affiliations:** 1 Division of Rheumatology, University of British Columbia, Vancouver, Canada; 2 British Columbia Cancer Agency, Vancouver, Canada; 3 Clinical Department of Pediatrics, Clinical Division of General Pediatrics, Medical University of Innsbruck, Innsbruck, Austria; 4 Section of Rheumatology, Nationwide Children's Hospital, Ohio State University, Columbus, OH, USA

## Abstract

The antinuclear antibody test (ANA) is a much overused test in pediatrics. The ANA does have a role in serologic testing but it should be a very limited one. It is often ordered as a screening test for rheumatic illnesses in a primary care setting. However, since it has low specificity and sensitivity for most rheumatic and musculoskeletal illnesses in children, it should not be ordered as a screening test for non-specific complaints such as musculoskeletal pain. It should only be used as a diagnostic test for children with probable Systemic Lupus Erythematosus (SLE) or Mixed Connective Tissue Disease, (MCTD) and other possible overlap-like illnesses. Such children should have developed definite signs and symptoms of a disease before the ANA is ordered. This review presents data supporting these conclusions and a review of the ANA literature in adults and children.

By limiting ANA testing, primary care providers can avoid needless venipuncture pain, unnecessary referrals, extra medical expenses, and most importantly, significant parental anxieties. It is best not to do the ANA test in most children but if it ordered and is positive in a low titer (<1:640), the results can be ignored if the child is otherwise well and does not have other features of a systemic illness.

## Background

Since the introduction of the indirect immunofluorescence (IF) test for antinuclear antibodies (ANA) by Friou in 1957 [1], ordering an ANA appears to have become a reflexive response to the question "could this patient have a rheumatic disease?" What is the evidence that ordering such tests is of any value, and what should be done with a positive test?

### The ANA test in health and disease

In diagnosing children with rheumatic disease, there are no markers as of now that identify a risk factor for a disease. Risk factors might allow primary prevention (e.g., screening for high serum cholesterol) or secondary prevention (e.g., detecting an illness before there are signs and symptoms e.g. pap smears for cervical cancer). In rheumatology only tertiary prevention is possible as illness may be detected as early in the disease course as possible in an effort to try and prevent the disease worsening and causing significant complications. Ideally there should be a screening test for children with arthritis or other positive rheumatic physical findings with a high specificity (test normal when someone does not have the disease) and high sensitivity (test abnormal when someone has the illness). A screening test should allow early diagnosis compatible with primary or secondary prevention.

For children with arthritis, only a few diagnostic tests are available (e.g., Lyme titer) and the ANA in particular has very limited usefulness as a diagnostic test. To put it another way, ideally a test should always be positive in those with a disease, and always negative in those without a disease. This situation rarely if ever occurs, but to be useful a test generally needs to have sensitivity and specificity of at least 90%. That is, at least 9 out of 10 individuals with the disease will have a positive test, a "true positive", and 9 out of 10 individuals without the disease will have a negative test. Unfortunately the ANA test, whether performed by IF or by an enzyme linked immunosorbent method (ELISA), fails to demonstrate these test characteristics [2]. The ELISA particularly has its problem with false positives.

Part of the problem is that the test is being used indiscriminately as part of "a rheumatologic work-up" or "rheumatology panel". No test can sensibly be expected to be accurate in the diagnoses of diseases as different as juvenile idiopathic arthritis (JIA), rheumatoid arthritis, SLE, MCTD, scleroderma, or the vasculitis diseases. Yet, in practice, this is what often seems to be asked of the ANA test. However, even if the test is used more sensibly to address specific questions such as: "does this child with a rash and fever have lupus?" or "does this child with a swollen knee have juvenile idiopathic arthritis (JIA)?", or "is this child with JIA going to develop uveitis?" we would argue that the ANA test is simply not accurate enough to answer even these questions.

### ANA in healthy populations

A number of studies have looked at the frequency of positive ANA tests in "healthy" individuals. A study by Arroyave et al. in 1988 [3] screened sera from 241 "normal" children, testing for only IgG ANA, using both mouse kidney and human epithelial cells (HEp-2 cells). The study found a maximum positivity rate of only 2.0% at the lowest dilutions. However, data from adult studies have found much higher rates. In an adult study from 15 international laboratories using HEp-2 cells as substrate [4], ANA positive tests occurred in 31.7% of a putatively normal population at a serum dilution of 1:40. Even at a dilution of 1:320, 3.3% of the sera were positive. Interestingly the ANA frequency did not differ significantly across the age range of 20-60 years. The rate of ANA positivity among blood donors in Holland was also quite high at 12.7%, with titers greater than 1:80 occurring in over 4% [5]. It is not clear why there is such a low frequency of ANA positivity in children, compared to the much higher frequency found in most adult studies (of which only two of many are referenced here). As ANA tests are usually performed on children or adults with musculoskeletal or rheumatologic symptoms or signs, the frequency of ANA among clinic populations is more pertinent to our discussion than the situation in the normal population

### ANA in clinic populations-ANA titers

Chudwin et al. in 1983 [6] evaluated the clinical and laboratory findings in 138 children with a positive ANA test. The authors interpreted the fact that two-thirds of the patients had a specific connective tissue disease as being indicative that the ANA test is useful. Yet the fact that one third did not have a definitive inflammatory disease indicates that the ANA test has a very high false positivity rate.

We evaluated the results of all the ANA tests performed at British Columbia's Children's Hospital over a 5 year span [7]. We found that the ANA test was positive at a titer of 1:20 or greater in 41% of all sera tested, and in 65% of all patients in whom a diagnosis could be obtained from the ordering physician. The frequency was the same for those children with or without a diagnosis of a rheumatic disease. At a screening serum dilution of 1:40 a positive test had a sensitivity of only 63% and a positive predictive value (the frequency that a positive test is indicative of disease) of only 33% for any rheumatic disease.

For SLE, MCTD or overlap syndrome, the ANA had a very high sensitivity of 98%, but a very low positive predictive rate of only 10%. Positive and negative predictive values are affected by the prevalence of the disease being tested. Therefore one might expect somewhat better predictive values from a pediatric rheumatology clinic than from a wide population of ill children. We concluded from this study that although a negative ANA test made the diagnosis of SLE or MCTD extremely unlikely, a positive test at even moderately high titers of 1:160 has little or no diagnostic value [7].

### ANA immunofluorescent staining

As our laboratory also provided information on the patterns of immunofluorescent staining, we have also been able to evaluate what further use this information might provide (previously unpublished data). Of 1369 individual patients sera tested, 445 were ANA positive in children with a known diagnosis. Of these children, 135 had a rheumatic disease (juvenile rheumatoid arthritis (JRA) (now known as JIA-the terms are used interchangeably here), SLE, MCTD, or juvenile dermatomyositis (JDM) but 310 had no convincing evidence of having a rheumatic disease.

Homogeneous, mitotic staining patterns were seen much more commonly in children with a rheumatic disease than those without (p = 0.001). Interestingly, a nucleolar pattern of staining was seen more commonly in children without a rheumatic disease (p = 0.03). This lack of an association of the nucleolar pattern in children with scleroderma specifically, and rheumatic disease in general has been commented on previously [8,9].

In our lab, no combination of ANA titer, or staining pattern was specific for any particular rheumatic disease. The test combinations with the best positive predictive values for a rheumatic disease were: a) a titer ≥1:640 with mitotic positive staining or b) a titer ≥1:640 with a homogenous and mitotic staining pattern. These tests had positive predictive values of 77% and 72% respectively. Yet these results were only slightly higher than the positive predictive value of 69% obtained with a titer of ≥1:640 alone, ignoring the pattern of staining. Although the addition of patterns somewhat increases the specificity and positive predictive value of the ANA test for a rheumatic disease, it does so at the expense of both the sensitivity and negative predictive value of the test using titers alone.

The pattern of staining also does not appear to be helpful in distinguishing between rheumatic diseases. For example, although high titer homogenous, mitotic positive staining was the most common combination seen in children with SLE, it was actually found in only 27.3% of ANA positive lupus patients. This combination was also found in 12.5% of ANA positive JRA and 5.4% of ANA positive JDM patients. This lack of specificity of the ANA immunofluorescent pattern has been recognized previously, both for adults and children [8,10,11]. A study by Parker et al. [11] evaluated the usefulness of combining ANA titer and pattern. Although they felt that knowledge of both titer and pattern was helpful, they did not calculate specific test characteristics, and in fact no combination was restricted to any single rheumatic disease. Our assessment of these data is that the addition of information about patterns of immunofluorescence does not appear to significantly improve the utility of the ANA test.

### Practical Concerns about ANA Testing

#### Referrals for positive ANA titers

As part of a study exploring what precipitated a referral to a pediatric rheumatologist, McGhee et al. [12] found that children referred, at least in part, because of a positive ANA test were no more likely to have a chronic inflammatory disease than children with a negative test.

In another study from a pediatric rheumatology clinic, only 55% of all of the children with a positive ANA test had an inflammatory rheumatic disease. Positive antibodies to double-stranded DNA or to extractable nuclear antigens (Sm, RNP) indicating SLE or MCTD were strongly correlated with an ANA titer ≥1:640. The authors recommended therefore, that these more specific tests be performed only if the child had a positive ANA test at high titer [13].

McGhee and colleagues reviewed the ANA titer clinical utility in 2004 [14]. One hundred and ten children were evaluated who had been referred for a positive ANA. Of the 110, 10 subsequently were found to have SLE, 18 had JRA, 1 had MCTD, and 1 had Raynaud's phenomenon. Neither a positive ANA neither titer nor the degree of positivity of the ANA helped pick out the children with SLE from the children without a chronic inflammatory disease. Tellingly, it wasn't the elevated ANA titer that distinguished the children with JRA from those with other musculoskeletal problems, but the history (e.g., morning stiffness) and the physical exam (e.g., presence of rash, swollen joints).

All these studies demonstrate that a positive ANA test is found frequently in a pediatric hospital population, and even in high titer has only a poor ability to determine whether a child has an inflammatory rheumatic disease.

#### Development of rheumatic diseases in ANA positive individuals

It could be argued that the finding of a positive ANA test indicates that the child has an occult disease that will become manifest later. Is there any evidence to support or refute this?

There is some evidence that ANA may sometimes precede the development of SLE by several years. Using Finland's Social Security Institution's population registry, Aho et al. [15] were able to trace 16 serum samples from apparently healthy subjects who later developed SLE or MCTD. Ten of the 16 (62.5%) samples were positive for ANA. Eight of the 11 (72.7%) were positive when the interval from sampling to onset of first symptoms was ≤ 2 years and 2 of 5 cases (40%) were positive when the interval was > 3 years. Based on an incidence rate of lupus of 5/100,000/year, the authors calculated that lupus would develop in less than one percent of the ANA positive individuals. Cabral et al. [16] followed the course of 24 children who were considered clinically not to have an inflammatory disease despite being ANA positive and found that no patient developed an overt inflammatory disease during a follow-up period of 61 months (range 13 to 138 months).

Some studies have evaluated the outcome in patients with fibromyalgia. Fibromyalgia is a musculoskeletal pain condition not thought to be an autoimmune disease. Interestingly, some fibromyalgia patients are also ANA positive, but there has been no evidence that the occurrence of a positive ANA influences patient outcome. Al-Allaf et al. [17] found the ANA positivity rate (titers not given, a positive result was simply defined as "plus") in their adult patients with fibromyalgia was 8.8%. This rate was almost identical to the 8.9% ANA positivity rate in their control patients with osteoarthritis. The 12 individuals who had fibromyalgia and were ANA positive were matched for age and sex with 12 ANA negative patients. Over a 2-4 year follow-up period one patient in the ANA positive group fulfilled criteria for SLE, and one in the ANA negative group fulfilled criteria for Sjögren's syndrome. The authors concluded that the ANA test (at least in low titer) was not a good predictor of future connective tissue disease.

In a separate study of 59 pediatric patients with fibromyalgia, 17 (28.8%) were ANA positive (mean titer 1:160). Fifty patients were followed for a mean of 18.3 months and during that time no patient developed a connective tissue disease [18]. We would conclude from these findings that only rarely is the presence of ANA the harbinger of occult SLE or another connective tissue disease.

#### ANA and other diseases

It should also be remembered that ANA are associated not only with the classical autoimmune diseases, but also with infection [19], malignancy [20,21] and drugs (Table 1) [22]. To illustrate this point, a study of Jones et al in 2006 analyzed 71 children who presented to a rheumatologist and who eventually were diagnosed with acute lymphocytic leukemia. Of the 71 children, 47 were tested for the ANA titer performed upon referral. Of the 47 ALL children, 8 (17%) had an elevated ANA titer [21]. Not only is the ANA test often positive in non-rheumatic diseases, it is often negative in many rheumatic diseases (Table 2).

**Table 1:** Diseases or syndromes that are frequently associated with an positive ANA test

Rheumatic	Non-rheumatic
Juvenile rheumatoid arthritis	Infection-(e.g., viral, Lyme)
Psoriatic arthritis	Malignancy (e.g., ALL)
Systemic lupus erythematosus	Environmental toxins
Systemic lupus erythematosus	Drugs
Juvenile dermatomyositis	
Mixed Connective Tissue Disease	
Drug-induced lupus syndrome	

Abbreviations ALL acute lymphocytic leukemia

**Table 2:** ANA positivity in rheumatic diseases in children

Diseases with ANA positivity	Diseases without ANA positivity
	
Systemic lupus erythematosus	Systemic onset JIA
Juvenile idiopathic arthritis (excluding enthesitis-related arthritis)	Rheumatic fever
Juvenile dermatomyositis	Post-streptococcal arthritis
Scleroderma-systemic and local	Enthesitis-related arthritis
Mixed Connective Tissue Disease	Reactive arthritis syndromes
	Henoch-Schönlein purpura
	Kawasaki disease
	Other vasculitis syndromes
	Sarcoidosis

Environmental toxins may also predispose to ANA production. Although hopefully not relevant in pediatrics, there is evidence of an increased frequency of ANA in individuals with silicone breast implants [23], and at least two studies have suggested that rural populations have a higher frequency of ANA positivity than urban populations, perhaps due to toxin exposure [24,25]. Therefore the finding of a positive ANA test should not blind the physician to the possibility of a non-autoimmune diagnosis.

A situation where a positive ANA test may be of some value is in children diagnosed with idiopathic thrombocytopenic purpura (ITP). In a study of 87 children with ITP, it was found that 36% of those with a positive ANA (titer ≥1:40) developed further "autoimmune symptoms". Five children developed SLE, compared to none of those who were ANA negative (p < 0.001). [26]

#### ANA positivity as a risk factor for uveitis in children with JIA

There is little doubt that in children with JIA the ANA test is more frequently positive in those with uveitis than in children without uveitis. The American Academy of Pediatrics recommends performing the ANA test as part of the screen for uveitis [27]. However, although there is a statistically significant difference between children with and without uveitis, we would argue that this difference itself is of little clinical significance.

In a recent study from Finland [28], for example, uveitis was found in 104 of 426 new cases of juvenile idiopathic arthritis. Antinuclear antibodies were found in 66% of those with uveitis compared to 37% of those without uveitis, a statistically significant difference. However if the presence or absence of a positive ANA test was used in determining the frequency of ophthalmologic examinations, it is possible that some of the 46/104 children with uveitis and negative ANA's might well have had a delayed diagnosis due to the partial reliance on the ANA positivity to determine the frequency of eye checkups. So there is risk for ANA negative children as well. This should not detract from the utility of a positive ANA in selecting out a population of children with arthritis who are at a higher risk for uveitis.

#### What should be done?

So what should be done with a positive ANA test? Our answer would be exemplified by the answer a local inhabitant gave when asked directions from place A to B by a foreign tourist: "I wouldn't be leaving from here!" In other words, it would be best if the ANA test had not been done in the first place! We would suggest that a positive ANA test can safely be ignored unless there are other suggestive clinical signs, and simple laboratory tests (such as a raised ESR or cytopenias) that point towards a diagnosis of lupus or similar connective tissue disease, particularly if the ANA titer is less than 1:640. Given the high false positive rate of ANA tests, a positive test cannot be used as confirmatory evidence that the child with a swollen joint has JIA, rather than some other serious condition such as septic arthritis, leukemia, or hemophilia. Similarly, symptoms such as fatigue and aches and pains in a child should not be ascribed to SLE simply because of a positive ANA test. It is much more likely that such a child has an idiopathic pain syndrome such as fibromyalgia or hypermobility. A negative ANA test is more useful than a positive one, as it does, for all practical purposes, exclude the diagnosis of SLE in a child.

What is needed is a cost-effectiveness study to evaluate whether the ANA test should be replaced by testing initially for anti-dsDNA and anti-ENA (anti-Sm, RNP, SSA and SSB) antibodies. Until that study is done, we would recommend that non-rheumatologists only do an ANA test if there is a fairly high probability (perhaps a 10+% chance) that a child's symptoms could be due to SLE or MCTD. If the test is positive at a titer of > 1:160 then it would be appropriate to order antibodies to dsDNA and ENA, with lower titers being ignored as well as complement levels.

We would strongly recommend that the ANA test is not ordered indiscriminately as part of "a rheumatologic work-up". This is not a new message. Other pediatric rheumatologists have pointed out in the literature that the ANA is a poor screening test and is being used inappropriately [7,9,12,14,16,29,30]. It is our hope that a continued demonstration of these facts will gradually decrease its inappropriate use. The cost of inappropriate referrals, extra venipuncture, unnecessary expense, and increased parental and child anxiety is a considerable problem that pediatric rheumatologists see every day.

## Conclusion

The question why the ANA test is so frequently positive in populations without an autoimmune disease remains a fascinating one. It suggests that the breaking of immunological tolerance is really quite common, but that this tolerance breakdown only rarely leads to disease. It is possible that antinuclear antibodies have some useful function that is not yet fully understood.

However, the ANA test has such a high false-positivity rate that a positive test is of little, if any, clinical utility as a screening test and should not be ordered routinely to screen children with musculoskeletal complaints. Its use should be limited to the diagnosis of SLE, MCTD, and similar systemic illnesses. If the test is performed, low titer ANA results (< 1:640) in most cases should be ignored unless the child is systemically ill and shows signs of SLE or a similar systemic disease.

## Abbreviations

ANA: antinuclear antibody titer; SLE or lupus: systemic lupus erythematosus; HEp-2 cells: human epithelial cells used as ANA substrate; ENA: extranuclear antigens; MCTD: mixed connective tissue disease; JRA: juvenile rheumatoid arthritis; [(JIA): also known as juvenile idiopathic arthritis]; JDM: juvenile dermatomyositis; dsDNA: double stranded DNA antibodies; Sm: Smith antigen (one of the extractable nuclear antigens); RNP: ribonuclear protein antigen; (another of the extractable nuclear antigens): SSA: Sjögren's syndrome A antigen; (Ro): (one more of the extractable nuclear antigens); SSB: Sjögren's syndrome B antigen; (La): (another of the extractable nuclear antigens); ITP: idiopathic thrombocytopenic purpura; (JRA): juvenile rheumatoid arthritis; (JIA): and juvenile idiopathic arthritis are the old and new classification terms for chronic arthritis diseases in children. JRA is used preferentially here as the data collection began before the term JIA was more universally accepted.

## Competing interests

The authors declare that they have no competing interests.

## Authors' contributions

PM conceived the project, acquired the data, and reviewed the results; MM did the statistical analyses. PM, MSH, and CHS were responsible for the interpretation of the data and literature as well as preparation of the manuscript. The final manuscript was approved by the authors.

## References

[B1] Friou GJ Fluorescent spot test for anti-nuclear antibodies Arthritis Rheum 1962 5 407 410 10.1002/art.1780050409 13895390

[B2] Giannini E Cassidy J, Petty R, Lindsley C, Laxer R Design, measurement, and analysis of clinical investigations Textbook of Pediatric Rheumatology 2005 5 Philadelphia: Elsevier and Saunders 142 173

[B3] Arroyava CM Giambrone MJ Rich KC Walaszek M The frequency of antinuclear antibody (ANA) in children by use of mouse kidney (MK) and human epithelial cells (HEp-2) as substrates J Allergy Clin Immunol 1988 82 741 4 10.1016/0091-6749(88)90073-5 3263996

[B4] Tan EM Feltkamp TEW Smolen JS Butcher B Dawkins R Fritzler MJ Gordon T Hardin JA Kalden JR Lahita RG Maini RN McDougal JS Rothfield NF Smeenk RJ Takasaki Y Wiik A Wilson MR Koziol JA Range of antinuclear antibodies in "healthy" individuals Arthritis Rheum 1997 40 1601 11 10.1002/art.1780400909 9324014

[B5] de Vlam K De Keyser F Verbruggen G Vandenbossche M Vanneuville B D'Haese D Veys EM Detection and identification of antinuclear autoantibodies in the serum of normal blood donors Clin Exp Rheumatol 1993 11 393 7 8403584

[B6] Chudwin DS Ammann AJ Cowan MJ Wara DW Significance of a positive antinuclear antibody test in a pediatric population Am J Dis Child 1983 137 1103 6 6356882 10.1001/archpedi.1983.02140370063021

[B7] Malleson PN Sailer M Mackinnon MJ Usefulness of antinuclear antibody testing to screen for rheumatic diseases Arch Dis Child 1997 77 299 304 10.1136/adc.77.4.299 9389231 PMC1717342

[B8] Osborn TG Patel NJ Moore TL Zuckner J Use of the HEp-2 cell substrate in the detection of antinuclear antibodies in juvenile rheumatoid arthritis Arthritis Rheum 1984 27 1286 9 10.1002/art.1780271111 6333875

[B9] Deane PMG Liard G Siegel DM Baum J The outcome of children referred to a pediatric rheumatology clinic with a positive antinuclear antibody test but without an autoimmune disease Pediatrics 1995 95 892 5 7761217

[B10] Wangel AG Teppo A-M Pollard A Howarth S Antibody profiles of sera giving different nuclear staining patterns Scand J Rheumatol 1984 13 303 9 10.3109/03009748409111300 6395320

[B11] Parker MD Kerby GP Combined titre and fluorescent pattern of IgG antinuclear antibodies using cultured cell monolayers in evaluating connective tissue diseases Ann Rheum Dis 1974 33 465 472 10.1136/ard.33.5.465 4608364 PMC1006307

[B12] McGhee JL Burks FN Sheckels JL Jarvis JN Identifying children with chronic arthritis based on chief complaints: absence of predictive value for musculoskeletal pain as an indicator of rheumatic disease in children Pediatrics 2002 110 354 9 10.1542/peds.110.2.354 12165590

[B13] Perilloux BC Shetty AK Leiva LE Gedalia A Antinuclear antibody (ANA) and ANA profile tests in children with autoimmune disorders: a retrospective study Clin Rheumatol 2000 19 200 3 10.1007/s100670050156 10870654

[B14] McGhee JL Kickingbird L Jarvis JN Clinical utility of ANA tests in children BMC Pediatrics 2004 4 13 10.1186/1471-2431-4-13 15245579 PMC476739

[B15] Aho K Koskela P Makitalo R Heliovaara M Palosuo T Antinuclear antibodies heralding the onset of systemic lupus erythematosus J Rheumatol 1992 19 1377 9 1433004

[B16] Cabral DA Petty RE Fung M Malleson PN Persistent antinuclear antibodies in children without identifiable inflammatory rheumatic or autoimmune disease Pediatrics 1992 89 441 4 1741219

[B17] Al Allaf AW Ottewell L Pullar T The prevalence and significance of positive antinuclear antibodies in patients with fibromyalgia syndrome: 2-4 years' follow-up Clin Rheumatol 2002 21 472 7 10.1007/s100670200118 12447630

[B18] Gedalia A Garcia CO Molina JF Bradford NJ Espinoza LR Fibromyalgia syndrome: experience in a pediatric rheumatology clinic Clin Exp Rheumatol 2000 18 415 9 10895386

[B19] Allen RC Dewez P Stuart L Gatenby PA Sturgess A Antinuclear antibodies using HEp-2 cells in normal children and in children with common infections J Paediatr Child Health 1991 27 39 42 10.1111/j.1440-1754.1991.tb00343.x 2043388

[B20] Swissa M Amital-Teplizki H Haim N Cohen Y Shoenfeld Y Autoantibodies in neoplasia. An unresolved enigma Cancer 1990 65 2554 8 10.1002/1097-0142(19900601)65:11<2554::AID-CNCR2820651126>3.0.CO;2-W 2337872

[B21] Jones OY Spencer CH Bowyer SL Dent PB Gottlieb BS Rabinovich CE A multicenter case-control study on predictive factors distinguishing childhood leukemia from juvenile rheumatoid arthritis Pediatrics 2006 117 e840 4 10.1542/peds.2005-1515 16651289

[B22] Byrne PA Williams BD Pritchard MH Minocycline-related lupus Br J Rheumatol 1994 33 674 6 10.1093/rheumatology/33.7.674 8019798

[B23] Cuellar ML Scopelitis E Tenenbaum SA Garry RF Silveira LH Cabrera G Espinoza LR Serum antinuclear antibodies in women with silicone breast implants J Rheumatol 1995 22 236 240 7738944

[B24] Rosenberg AM Semchuk KM McDuffie HH Ledingham DL Cordeiro DM Cessna AJ Irvine DG Senthilselvan A Dosman JA Prevalence of antinuclear antibodies in a rural population J Toxicol Environ Health A 1999 57 225 236 10.1080/009841099157674 10406347

[B25] Spiewak R Stojek N Antinuclear antibodies among eastern-Polish rural inhabitants Ann Agric Environ Med 2003 10 207 9 14677913

[B26] Zimmerman SA Ware RE Clinical significance of the antinuclear antibody test in selected children with idiopathic thrombocytopenic purpura J Pediatr Hematol Oncol 1997 19 297 303 10.1097/00043426-199707000-00006 9256827

[B27] Cassidy J Kivlin J Lindsley C Nocton J Ophthalmologic examinations in children with juvenile rheumatoid arthritis. The Section on Rheumatology and the Section on Ophthalmology Pediatrics 2006 117 1843 45 10.1542/peds.2006-0421 16651348

[B28] Kotaniemi K Kautiainen H Karma A Aho K Occurrence of uveitis in recently diagnosed juvenile chronic arthritis: a prospective study Ophthalmol 2001 108 2071 75 10.1016/S0161-6420(01)00773-4 11713082

[B29] Siegel DM Antinuclear antibody (ANA) testing Pediatrics in Review 2003 24 320 1 10.1542/pir.24-9-320 12949270

[B30] Jarvis J Commentary-ordering lab tests for suspected rheumatic disease Pediatric Rheumatology Online Journal 2008 6 19 23 10.1186/1546-0096-6-19 19014701 PMC2588570

